# Microenvironment generated during EGFR targeted killing of pancreatic tumor cells by ATC inhibits myeloid-derived suppressor cells through COX2 and PGE_2_ dependent pathway

**DOI:** 10.1186/1479-5876-11-35

**Published:** 2013-02-09

**Authors:** Archana Thakur, Dana Schalk, Elyse Tomaszewski, Sri Vidya Kondadasula, Hiroshi Yano, Fazlul H Sarkar, Lawrence G Lum

**Affiliations:** 1 Departments of Oncology and Medicine, Wayne State University and Barbara Ann Karmanos Cancer Institute, Detroit, MI 48201, USA; 2 Department of Pathology, Wayne State University and Karmanos Cancer Institute, Detroit, MI 48201, USA; 3 Immunology and Microbiology, Wayne State University and Karmanos Cancer Institute, Detroit, MI 48201, USA

**Keywords:** 3D culture model, Pancreatic cancer, Activated T-cells, Bispecific antibody, Epidermal growth factor receptor, Myeloid derived suppressor cells

## Abstract

**Background:**

Myeloid-derived suppressor cells (MDSCs) are one of the major components of the immune-suppressive network, play key roles in tumor progression and limit therapeutic responses. Recently, we reported that tumor spheres formed by breast cancer cell lines were visibly smaller in a Th_1_ enriched microenvironment with significantly reduced differentiation of MDSC populations in 3D culture. In this study, we investigated the mechanism(s) of bispecific antibody armed ATC mediated inhibition of MDSC in the presence or absence of Th_1_ microenvironment.

**Methods:**

We used 3D co-culture model of peripheral blood mononuclear cells (PBMC) with pancreatic cancer cells MiaPaCa-2 [MiaE] and gemcitabine resistant MiaPaCa-GR [MiaM] cells to generate MDSC in the presence or absence of Th_1_ cytokines and EGFRBi armed ATC (aATC).

**Results:**

We show significantly decreased differentiation of MDSC (MiaE, p<0.005; MiaM, p<0.05) in the presence of aATC with or without Th_1_ cytokines. MDSC recovered from control cultures (without aATC) showed potent ability to suppress T cell functions compared to those recovered from aATC containing co-cultures. Reduced accumulation of MDSC was accompanied by significantly lower levels of COX2 (p<0.0048), PGE_2_ (p<0.03), and their downstream effector molecule Arginase-1 (p<0.01), and significantly higher levels of TNF-α, IL-12 and chemokines CCL3, CCL4, CCL5, CXCL9 and CXCL10 under aATC induced Th_1_ cytokine enriched microenvironment.

**Conclusions:**

These data suggest aATC can suppress MDSC differentiation and attenuation of their suppressive activity through down regulation of COX2, PGE_2_ and ARG1 pathway that is potentiated in presence of Th_1_ cytokines, suggesting that Th_1_ enriching immunotherapy may be beneficial in pancreatic cancer treatment.

## Background

Most cancers can evade the immune surveillance and circumvent antitumor immune defenses by several passive and active mechanisms. Preclinical and clinical studies suggest that regulatory/suppressor immune cells in the inflammatory tumor microenvironment can induce an immune tolerizing effect and inhibit the ability of immune based therapies or cancer vaccines to initiate robust anti tumor immune responses [1,2]. Among many suppressor regulators, myeloid-derived suppressor cells (MDSCs) are of great interest because they have the capacity to suppress both the adaptive immune response mediated by CD4^+^ and CD8^+^ T cells [3-5] and the cytotoxic activities of natural killer (NK) and NKT cells [6].

Increasing evidence suggests that tumor- and MDSC-derived arachidonic acid metabolites, cyclooxygenase-2 (COX2) and prostaglandin E_2_ (PGE_2_) play critical roles in T cell suppression [7-11]. One of the mechanisms of COX2 and PGE_2_ mediated suppression of T cells is through the induction of arginase-1 (ARG1) [12,13]. A better understanding of these molecules in the tumor microenvironment and assessment of the regulatory cross talk between tumor cells and the immune cells would help in developing clinically effective immunotherapeutic approaches against pancreatic cancer.

In our previous study in breast cancer 3D culture model, we reported a significant reduction of MDSC in the presence of Th_1_ cytokines and activated T cells armed with anti-CD3 x anti-Her2 bispecific antibodies (aATC) [14]. In this study, we investigated the mechanism(s) of aATC mediated inhibition of MDSC in the presence or absence of Th_1_ microenvironment. Furthermore, we examined whether presence of aATC in the tumor microenvironment can shift the immune suppressive tumor microenvironment to immune activating anti-tumor Th_1_ microenvironment.

## Methods

### Cell lines

The human pancreatic cancer (PC) cell lines (MiaPaCa-2 cells with epithelial characteristics [MiaE] and gemcitabine resistant MiaPaCa-GR cells with mesenchymal characteristics [MiaM]) were maintained in DMEM culture media (Lonza Inc., Allendale, NJ) supplemented with 10% FBS (Lonza Inc.), 2 mM L-glutamine (Invitrogen, Carlsbad, CA), 50 units/ml penicillin, and 50 μg/ml streptomycin (Invitrogen). MiaM was maintained in 200 nM gemcitabine in DMEM media. MiaE show typical epitheloid like morphology whereas MiaM show mesenchymal like morphology. The reason for using chemo sensitive and resistant pancreatic cell lines was to evaluate whether ATC armed with bispecific antibodies can be effective, irrespective of chemo resistance of the cell lines. Both cell lines showed high expression of EGFR by flow cytometry (data not show).

### Expansion and generation of ATC

CD3^+^ T cells from PBMC were activated and expanded using 20 ng/ml of OKT3 and 100 IU/ml of IL-2 for 14 days at a concentration of 1–2 × 10^6^ PBMC/ml in RPMI-1640 supplemented with 10% FBS [15].

### Production of anti-OKT3 x anti-EGFR bispecific antibodies (EGFRBi)

Bispecific Antibodies (BiAb) were produced by chemical heteroconjugation of OKT3 (a murine IgG2_a_ anti-CD3 monoclonal antibody, Ortho Biotech, Horsham, PA) and Erbitux (a humanized anti-EGFR IgG_1_, Genentech Inc., San Francisco, CA) as described [15,16]. ATC were armed with EGFRBi (aATC) using a previously optimized concentration of BiAb of 50 ng/10^6^ ATC.

### 3D culture in matrigel

Cells were prepared at a concentration of 2,500 cells/ml in RPMI-1640 or DMEM culture media. Single cells are overlaid on a solidified layer of Matrigel measuring approximately 1 mm in thickness as described [14]. Briefly, wells were coated with 100% Matrigel in 0.25-ml aliquots in 24-well glass bottom plates and allowed to solidify by incubating at 37°C for 30 min. Pancreatic cancer cells were then seeded onto the matrigel base as a single-cell suspension in the medium containing 2% matrigel, in the presence or absence of Th_1_ cytokines (10 ng/ml IFN-γ and 100 IU/ml IL-2). PBMC were added either simultaneously or after 5–7 days when tumor spheres were formed, PBMC were added at 10:1 ratio (10 PBMC:1 tumor cell). EGFRBi aATC were added after 7 days of tumor cell and PBMC 3D co-culture at 10:1 (10 aATC/1 tumor cell) ratio. The medium was replaced every 4 days. Tumor spheres were visualized in 5–7 days in 3D culture.

### Cytotoxicity assay

Tumor cells were seeded in 24-well plate at 100,000 cells/well in volume of 1 ml. Cells were allowed to adhere followed by incubation with aATC for 3–5 days at 1:1 E:T in the presence or absence of Th_1_ cytokines. At the end of incubation, 3-(4,5-dimethylthiazolyl-2)-2,5-diphenyltetrazolium bromide (MTT) was added (40 μL/well of 5 mg/mL MTT in PBS) to each well and incubated in the dark for 3 h at 37°C. After removal of the medium, the dye crystals formed in viable cells were dissolved in isopropanol and detected by reading the absorption at 595 nm in the Tecan Ultra plate reader. Experiments were repeated three times in quadruplicate wells to ensure reproducibility.

### Flow cytometry to identify MDSC

Flowcytometry was done at the Microscopy, Imaging and Cytometry Resources Core at Karmanos Cancer Institute, Wayne State University. The phenotype of MDSC generated in 3D co-culture of tumor cells with PBMC was evaluated for expression of CD33, CD11b, CD14 and HLA-DR. After non-adherent cells were collected, matrigel was digested to collect tumor cells or tumor associated MDSC and, washed with FACS buffer (0.2% BSA in PBS). Cells collected prior to digestion were pooled with matrigel digested single cell suspension before staining. Cells were stained for 30 min on ice with mixtures of fluorescently conjugated mAbs or isotype-matched controls, washed twice with FACS buffer and analyzed. Antibodies used for staining include: anti-CD11b, -CD14, -CD15, -CD33, -HLA-DR, (BD Biosciences San Jose, CA). Cells were analyzed on a FACScalibur (BD Biosciences) and data were analyzed using CellQuest software (BD Biosciences). Cells were gated on CD11b^+^/CD33^+^ population and analyzed for CD14^+^/HLA-DR^-^ and CD15^+^/HLA-DR^-^ expression.

### MDSC isolation and co-culture with T cells

Cells were collected from the digested matrigel cultures. CD33^+^ cells were isolated from each culture using anti-CD33 magnetic microbeads (Miltenyi Biotec) as previously described [14]. The purity of isolated cell populations was found to be >80% by flow cytometry. Effect of MDSC on T cell proliferation was measured by co-incubation of CD33^+^ cells with purified CD3^+^ T-cells. Briefly, purified CD3^+^ T-cells plated at 0.5 7 × 10^5^ cells/well in 24-well plates coated either with anti-CD3 antibodies (0.5 μg/ml in PBS) or isotype matched control antibodies. Irradiated (2500 rads) CD33^+^ cells were then added at various MDSC: T cells or ATC ratios ranging from 1:5–1:20 in a final volume of 500 μl of medium. Control wells did not receive any MDSC. The plates were incubated for 24–72 hrs (for CD3+ T cells) or 4 hrs (for ATC) at 37°C in humidified 5% CO_2_ atmosphere followed by a flow cytometric analysis of T cell activation and function using anti-CD71, anti-CD62L, anti-NKG2D and anti-IFN-γ antibodies.

### Inhibition of T cell proliferation and cytotoxicity by MDSC

Inhibitory activity of the CD33^+^ cells isolated from matrigel co-cultures towards T cell proliferation and cytotoxicity was examined as described previously (14).

### Flow cytometric analysis for COX2 and arginase-1 positive cells

Co-cultures were evaluated for the expression of, COX2 and Arginase-1 (ARG1) using anti-ARG1-PE, and anti-COX2-FITC antibodies along with anti-CD11b, -CD14, -CD15, -CD33, -HLA-DR (BD Biosciences San Jose, CA) in a 7-color analysis by FACScalibur (BD Biosciences). Data were analyzed using FloJo software. Total COX2 or ARG1 positive cells as well as ARG1 or COX2 positive MDSC were analyzed. Cells were gated for CD33^+^/CD11b^+^/HLA-DR^-^ and analyzed for CD14 or CD15 versus ARG1, CD14 or CD15 versus COX2 expression.

### PGE_2_ production

Analysis of PGE_2_ was performed by EIA kit as per manufacturer’s instruction (Enzo Life Sciences, Plymouth Meeting, PA) in the culture supernatants from 3D co-cultures.

### Cytokine profiling of co-cultures

Cytokines were quantitated in culture supernatants collected from matrigel co-cultures in the presence or absence of Th_1_ cytokines and in the presence or absence of ATC or armed ATC using a 25-plex human cytokine Luminex Array (Invitrogen, Carlsbad, CA) on a Bio-Plex system (Bio-Rad Lab., Hercules, CA). The limit of detection for these assays is < 10 pg/mL based on detectable signal of > 2 fold above background (Bio-Rad). Cytokine concentrations were automatically calculated by the BioPlex Manager Software (Bio-Rad).

### Statistical analysis

Quantitative data are presented as the mean of at least three or more independent experiments ± standard deviation. A one-way ANOVA was used to determine whether there were statistically significant differences among different conditions within each experiment. Differences between groups were tested via an unpaired, two-tailed *t* test.

## Results

### Armed ATC induced Th_1_ cytokine microenvironment inhibits MDSC differentiation

**Figure 1:**
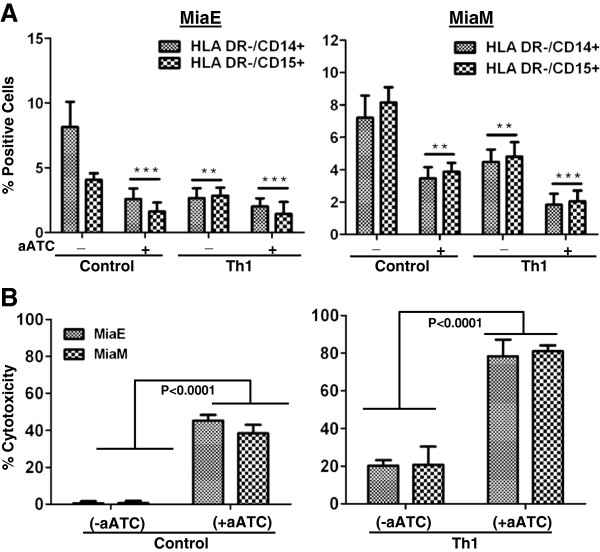
**Effect of Th**_**1 **_**cytokines on pancreatic cancer cells. A**) Shows reduced percentage of monocytic CD33^+^/CD11b^+^/CD14^+^/HLA-DR^-^ and granulocytic CD33^+^/CD11b^+^/CD15^+^/HLA-DR^-^ MDSC populations in the presence of aATC for MiaE (p<0.0021) and MiaM (p<0.046) in the presence of aATC or aATC+Th_1_ cytokines [MiaE (p<0.00041) and MiaM (p <0.0002)] compared to control co-cultures (n=3). **B**) Shows that increased cytotoxicity by EGFRBi armed ATC after 3 days at an E:T ratio of 1:1 when grown in the presence of Th_1_ cytokines.

Consistent with our earlier studies [14], proportions of monocytic CD33^+^/CD11b^+^/CD14^+^/HLA-DR^-^ and granulocytic CD33^+^/CD11b^+^/CD15^+^/HLA-DR^-^ MDSC populations were reduced in the presence of aATC for both MiaE (p<0.00021) and MiaM (p<0.0046) in the presence of aATC compared to control co-cultures. Reduction in CD33^+^/CD11b^+^/CD14^+^/HLA-DR^-^ and CD33^+^/CD11b^+^/CD15^+^/HLA-DR^-^ MDSC populations were highly significant in MiaE (p<0.00041) and MiaM (p <0.0002) when both aATC and Th_1_ cytokines were added to co-cultures (Figure 1A). These data suggest that the microenvironment induced by interactions of aATC with tumor cells is inhibitory for MDSC differentiation and this effect was more pronounced in a Th_1_ cytokine enriched microenvironment (n=3). Figure 1B shows that tumor cells become more susceptible for EGFRBi armed ATC mediated killing when grown in the presence of Th_1_ cytokines.

### MDSC mediated suppression of T cell proliferation and cytotoxic activity was partially reversed by EGFRBi armed ATC

**Figure 2:**
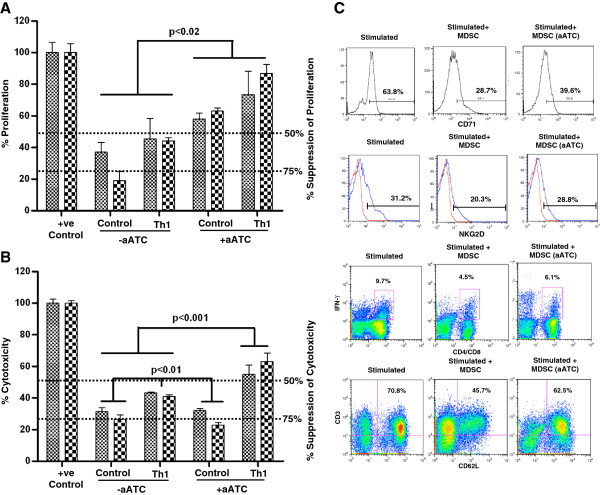
**aATC can attenuate the suppressive properties of MDSC.** CD33^+^ cells isolated either from control condition or from co-cultures containing aATC were added to cytotoxicity assay, proliferation assay and anti-CD3 stimulated T cells for 24-72 h. **A**) Shows the suppressive effect of CD33^+^ MDSC on anti-CD3-stimulated autologous T-cell proliferation. Proliferation was significantly suppressed by more than 50% in the presence of CD33^+^ MDSC isolated from control co-cultures, and this suppression was reversed if CD33^+^ MDSC were isolated from aATC containing co-cultures. **B**) Shows the suppressive effect of CD33^+^ MDSC on aATC mediated cytotoxicity. **C**) Top panel, right histogram shows CD71 expression on stimulated CD3^+^ T cells (positive control); middle histogram shows suppressive effect of CD33^+^ MDSC isolated from control co-cultures (without aATC) and right histogram show attenuated suppressive effect on CD71 expression when CD33^+^ cells were isolated from co-cultures that contained aATC. Second panel, right histogram shows NKG2D expression on stimulated CD3^+^ NK T cells (positive control); middle histogram shows the suppressive effect of CD33^+^ MDSC isolated from control co-cultures (without aATC) and right histogram shows the attenuated suppressive effect on NKG2D expression when CD33^+^ cells were isolated from co-cultures that contained aATC on anti-CD3-stimulated T-cells. Third panel, right histogram shows IFN-γ positive T cells upon stimulation with PC cells (positive control); middle histogram show suppressive effect of CD33^+^ MDSC isolated from control co-cultures (without aATC) on IFN-γ production and right histogram show attenuated suppressive effect on IFN-γ production when CD33^+^ cells isolated were from co-cultures that contained aATC. Bottom panel, right histogram shows CD62L expression (migration marker) on naive T cells (positive control); middle histogram show suppressive effect of CD33^+^ MDSC isolated from control co-cultures (without aATC) and right histogram show attenuated suppressive effect on CD62L expression when CD33^+^ cells added were isolated from co-cultures that contained aATC.

CD33^+^ MDSC isolated from various co-culture conditions were incubated with OKT3 stimulated T cells at 1:5 ratio. T cell proliferation was suppressed by more than 50% in the presence of CD33^+^ cells isolated from cultures without aATC. However, CD33^+^ MDSC isolated from aATC containing co-cultures showed significantly reduced capacity to inhibit proliferation of T cells (p<0.02). Likewise, the cytotoxicity mediated by aATC directed at SK-BR-3 targets was inhibited by 70% at 1:10:2 ratio (Tumor cell:aATC:CD33^+^) after adding CD33^+^ cells isolated from control conditions. The inhibitory effect of CD33^+^ cells on T cell cytotoxicity was significantly attenuated (p<0.001) in the presence of Th_1_ cytokines (n=3; Figure 2A and B).

### MDSC mediated suppression of activated T and NK cells was partially reversed by aATC

T cells from three different culture conditions were stained for T cell activation markers CD71 (Upper panel) and NKG2D (Lower panel at the end of 72 h co-cultures). OKT3 stimulated T cells showed 63.8% CD71 positive cells, this was considered as 100% positive control. CD71 expression was suppressed by 55% in the presence of CD33^+^ cells (isolated from co-cultures without aATC). This suppression was partially reduced to 38% when T cells were incubated with CD33^+^ cells isolated from aATC containing co-cultures (n=3; Figure 2C, top panel). Immunostaining for NKG2D showed 31.2% of T cells positive for NKG2D (Positive control, 100%) and this expression was decreased to 20.3% (35% inhibition from control) by the addition of CD33^+^ cells isolated from co-cultures without aATC. This inhibition was reversed by adding CD33^+^ cells isolated from aATC containing co-cultures, restoring the expression of NKG2D to 28.8% (n=3; Figure 2C, second panel). These data suggest that aATC inhibited the immune suppressive ability of MDSC.

### MDSC mediated suppression of T cell IFN-γ production was reversed by aATC

We asked whether addition of MDSC to ATC would suppress the ability of ATC to produce IFN-γ when stimulated with MiaE targets at a 1:10:2 ratio (Tumor cell:ATC:CD33^+^) for 4 hrs. Stimulated ATC showed 9.7% cells positive for intracellular IFN-γ (positive control, 100%). Incubation of stimulated ATC with CD33^+^ cells isolated from co-cultures without aATC inhibited IFN-γ production by 54%. This inhibition was partially reverted when ATC were mixed with CD33^+^ cells isolated from aATC containing co-cultures to 37% (n=3; Figure 2C, third panel).

### MDSC mediated suppression of CD62L expression on naive T cells was reversed by aATC

Since, MDSC have been shown to mitigate the expression of L-selectin (CD62L) on naïve T cells [17], we asked whether incubation of T cells with MDSC isolated from various culture conditions can alter the expression of CD62L differentially. Naïve T cells showed 70.8% expression of CD62L (positive control, considered 100%), CD62L expression was inhibited by 65% in the presence of CD33^+^ cells (isolated from co-cultures without aATC). CD33^+^ isolated from aATC containing co-cultures showed significantly reduced suppression of CD62L expression (12%) on naive T cells (n=3; Figure 2C, bottom panel).

### Armed ATC mediated microenvironment inhibits MDSC differentiation by suppressing MDSC-associated suppressive factors

**Figure 3:**
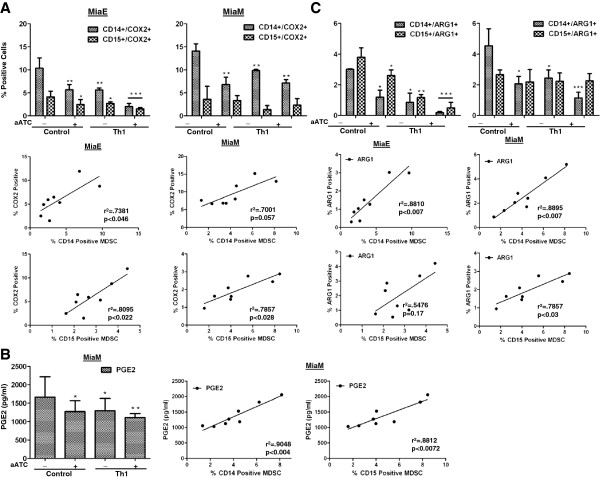
**Increased accumulation of MDSC is associated with increased levels of lipid mediators and ARG1. A**) Shows accumulation of COX2^+^ cells in co-cultures containing MiaE or MiaM cells in the presence or absence of aATC or aATC+Th_1_ cytokines. Bottom panel show a correlation between COX2 positive cells and frequency of monocytic and granulocytic MDSC in co-cultures for both cell lines. **B**) Shows PGE_2_ levels in the supernatants from co-cultures containing MiaM cells in the presence or absence of aATC or aATC+Th_1_ cytokines. Bottom panel show a strong correlation between PGE_2_ levels and frequency of monocytic and granulocytic MDSC in co-cultures for MiaM cells. **C**) Shows presence of ARG1^+^ cells in co-cultures containing MiaE or MiaM cells in the presence or absence of aATC or aATC+Th_1_ cytokines. Bottom panel show a correlation between of ARG1 positive cells and frequency of monocytic and granulocytic MDSC in co-cultures for both cell lines. */**/*** signifies statistically significant differences (*P < 0.05, **P < 0.01, ***p<0.001).

Previous studies have shown that COX2, PGE_2_, and ARG1 axis plays a critical role in MDSC development [12,18], and that ARG1 activity is accompanied by decreased expression of CD3ζ and diminished production of IFN-γ in activated T cells [13,19,20]. We investigated whether EGFRBi armed ATC modulate the COX2, PGE_2_ and ARG1 pathway leading to the inhibition of MDSC function. Addition of EGFRBi aATC in co-cultures exhibited significantly reduced numbers of COX2^+^ (MiaE, p<0.0045; MiaM, p<0.0048) monocytic MDSCs compared to control co-cultures without aATC (Figure 3A). Since COX2 is the key enzyme regulating PGE_2_ synthesis, we measured PGE_2_ in the culture supernatants of MiaM cells. PGE_2_ levels were significantly reduced (MiaM, p<0.03) in co-cultures containing EGFRBi aATC (Figure 3B). Next, we determined the ARG1^+^ cells (Figure 3C) in co-culture, which followed the same pattern as COX2 and PGE_2_ showing significantly reduced numbers in co-cultures containing EGFRBi aATC and MiaE (p<0.01) or MiaM (p<0.05). Reduction in COX2, ARG1 and PGE_2_ levels were highly significant in co-cultures of MiaE (p<0.0005) and MiaM (p<0.005) in the presence of both aATC and Th_1_ cytokines. More interestingly, there was a strong correlation between the COX2, PGE_2_ and ARG1 expression and the accumulation of monocytic (CD14^+^) or the granulocytic (CD15^+^) MDSC Lower panels of Figure 3A, 3C and left panels of 3C. These data suggest that aATC induced Th_1_ cytokines may inhibit the tumor- and MDSC-derived immunosuppressive factors in the tumor microenvironment. aATC mediated inhibition of MDSC activity was more potent in the presence of IL-2 and IFN-γ.

### Armed ATC induce cytokines and chemokines that are suppressive for MDSC differentiation and activation

**Figure 4:**
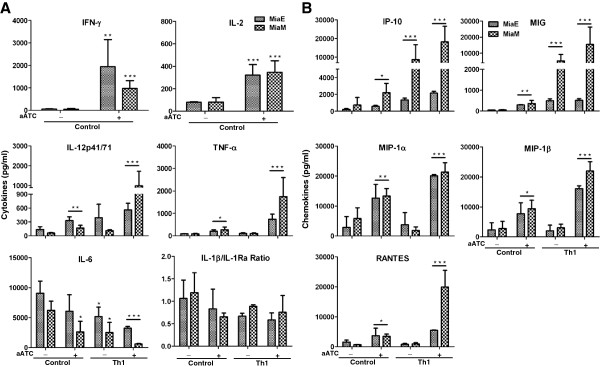
**Cytokine profile of culture supernatants measured by multiplex luminex system. A**) shows the increased levels of cytokines IL-6 and IL-1β/IL-1Ra ratio, and low levels of cytokines IFN-γ, IL-2, IL-12 and TNF-α in control co-cultures (without aATC or Th_1_ cytokines) compared to culture supernatants containing aATC or aATC and Th_1_ cytokines. **B**) Shows low levels of CXCL10/IP-10, CXCL9/MIG, CCL3/MIP-1α, CCL4/MIP-1β and CCL5/RANTES expression in control co-cultures (without aATC or Th_1_ cytokines) compared to culture supernatants containing aATC or aATC and Th_1_ cytokines. */**/*** signifies statistically significant differences (*P < 0.05, **P < 0.01, ***p<0.001).

We recently reported that in Th_1_ cytokine enriched microenvironment, MIG/CXCL9 and IP-10/CXCL10 were upregulated while IL-1β and IL-6 were downregulated with concomitant reduction in the percentage of MDSC in a 3D breast cancer model [14]. Consistent with these observations, either aATC or aATC in Th_1_ cytokine containing co-cultures showed significantly lower levels of proinflammatory cytokine IL-6 and higher IL-1β/IL-1Ra ratio compared to control conditions without aATC or aATC and Th_1_ cytokines. On the other hand, levels of IFN-γ, IL-2, IL-2R and IL-12p41/71 were significantly higher in culture supernatants from aATC or aATC and Th_1_ cytokines containing co-cultures compared to control condition (Figure 4A). Likewise, chemokines that are known to suppress MDSC differentiation and activation such as CXCL9/IP-10 and CXCL9/MIG were significantly higher in culture supernatants either from aATC or aATC and Th_1_ cytokines containing co-cultures compared to control condition (Figure 4B). High levels of IFN-γ, IL-2, IL-12, CXCL9 and CXCL10 corroborate with reduced number of MDSC.

## Discussion

Recently, we reported that tumor spheres formed by breast cancer cells were visibly smaller in size in a Th_1_ enriched microenvironment, differentiation of granulocytic CD14^−^/HLA-DR^−^/CD11b^+^/CD33^+^ and monocytic CD14^+^/HLA-DR^−^/CD11b^+^/CD33^+^ MDSC populations was reduced with further reduction and attenuation of their suppressive activity in the presence of aATC [14]. In this study, we investigated the mechanism(s) of aATC mediated inhibition of MDSC in the presence or absence of Th_1_ microenvironment. We show significantly decreased differentiation and accumulation of MDSC in the presence of aATC or aATC and Th_1_ cytokines. The decreased percentage of MDSC was paralleled by significantly lower levels of IL-6, COX2, PGE_2_, ARG1 in the presence of aATC or aATC and Th_1_ cytokines. While, levels of IFN-γ, IL-2, TNF-α, IL-12 and chemokines CXCL9 and CXCL10 were higher in the presence of aATC or aATC and Th_1_ cytokines.

Consistent with other studies that inflammation is associated with the expansion of MDSC [21,22], our data also show that increased numbers of MDSC were accompanied by increased levels of proinflammatory cytokines IL-6 and IL-1β/IL-1Ra ratio. A delayed accumulation of MDSC and reduced primary and metastatic tumor progression was reported in mice that have reduced inflammation due to IL-1 receptor-deficiency [23,24]. On the other hand, excessive inflammation in IL-1R antagonist-deficient mice promoted the accumulation of MDSC and produced MDSC with enhanced suppressive activity [23,24]. Relevance of increased levels of TNF-α in the presence of Th_1_ cytokines or Th_1_ cytokines + aATC in the context of MDSC is not clear. TNF has been shown to play a crucial role in the differentiation of myeloid cells [25,26]. However, binding of TNF to TNFR-1 and TNFR-2 activates distinct signaling pathways [27-30]. Depending on TNF signaling pathway it may favor tumor growth and differentiation of MDSC or may induce immune responses [31].

In addition to cytokines, bioactive lipid mediators, such as PGE_2_ and COX2 produced by many tumors are known to induce the inflammatory and immune suppressive tumor microenvironment [10,32-34]. Kalinski et al. showed that PGE_2_ can modulate the Th_1_ responses by impairing IL-12, and IFN-γ expression [35-37]. PGE_2_ and COX2 amplify ARG1 levels in MDSC and suppress the adaptive immune response in part through ARG1 production that enhances the L-arginine catabolism and thus depletion of L-arginine [13,19,38,39]. Catabolism of L-arginine is essential for the suppressive activity of MDSC, which serves as a substrate for two enzymes, oxide synthase (iNOS) and arginase 1 (ARG1). MDSCs express high levels of both ARG1 and iNOS and both these enzymes play roles in the inhibition of T-cell function [13,19,20]. Depletion of L-arginine in the tumor microenvironment leads to the inhibition T cell proliferation by decreasing expression of the CD3ζ chains [19]. and induction of T cell apoptosis [40]. Collectively, these studies show a strong association between expansion of MDSCs and inflammation mediated by the arachidonic acid cascade. Consistent with these findings, our data suggest a strong correlation between increased accumulation of MDSC and high levels of COX2/PGE_2_/ARG1 expression.

Analysis of chemokines showed significantly reduced levels of CCL3/MIP1α, CCL4/MIP-1β, CCL5/RANTES, CXCL9/MIG and CXCL10/IP-10 in the supernatants from control culture conditions (without aATC) which increased dramatically when either aATC or aATC and Th_1_ cytokines were added to the co-cultures. Co-cultures with reduced chemokine levels contained a significantly higher percentage of MDSC and significantly higher levels of COX2 and PGE_2_. PGE_2_ has been shown to inhibit mRNA and protein expression of chemokines including CCL3/MIP1α, CCL4/MIP-1β, CXCL10/IP-10 in activated monocytes and macrophages [41-44]. COX2 and PGE_2_ were reported to deregulate the chemokine production of DCs, abrogating the CXCL9/MIG, CXCL10/IP-10 and CCL5/RANTES-mediated ability of DC to attract naive, effector, and memory T and NK cells [42,44-46].

Activated T cells express a variety of surface markers, including CD25, CD71, CD95, CD137, HLA-DR, and secrete Th_1_ cytokines IL-2 and IFN-γ [47]. We analyzed CD71, CD62L and IFN-γ as T cell activation and functional markers and NKG2D as NK or NKT cell activation marker to assess the suppressive activity of MDSC on T cells and NK cells. In the presence of MDSC isolated from control (without aATC), the expression of all the T cell activation markers were markedly downregulated whereas MDSC isolated from aATC containing co-cultures showed attenuated inhibition of T cells activation markers. Study by Ochoa et al. showed restoration of IFN-γ production and T-cell proliferation after MDSC depletion [12]. MDSC can abrogate the expression of L-selectin (CD62L) on both CD4^+^ and CD8^+^ T cells, subverting the homing of these cells to the tumor site leading towards a dominant immunosuppressive microenvironment [17].

## Conclusion

**Figure 5:**
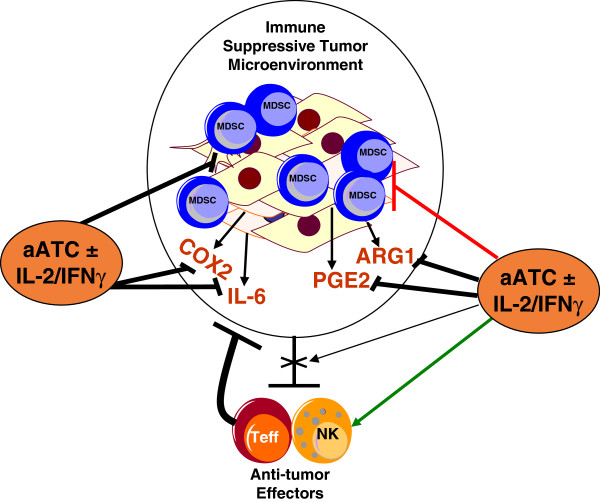
**Immune suppressive microenvironment can partially be reverted with aATC immunotherapy.** A schematic summary of the present study showing high levels of COX2/PGE_2_/ARG1 in the tumor microenvironment, which promote tumor growth and MDSC development and maintain immune suppressive microenvironment inhibiting proliferation and activation of T cells and NK cells. aATC induced Th_1_ cytokines may inhibit the tumor- and MDSC-derived immunosuppressive factors COX2, PGE_2_, ARG1 and IL-6 in the tumor microenvironment and may restore the immune activating microenvironment.

We show that EGFRBi aATC can target both pancreatic tumor cells and its microenvironment. EGFRBi aATC: 1) can efficiently kill tumor cells; 2) EGFRBi aATC may disable the COX2 and PGE_2_ mediated suppression of CTLs, Th_1_, and NK cells by modulating immune suppressive microenvironment to immune activating Th_1_ microenvironment. Our data suggest that cellular immunotherapy using aATC with low levels of IL-2 and IFN-γ will not only target the tumor cells but may reverse the suppressive tumor environment to allow recruitment of CTLs and NK cells at the tumor sites and may induce an endogenous anti-tumor immune responses (Figure 5).

## Competing interests

The authors declare that they have no competing interests.

## Authors’ contributions

AT conceived and designed the study, performed statistical analysis and wrote the manuscript. DLS performed the experiments and participated in the data analysis. ENT, SVK and HY helped in carrying out the immunoassays. FHS and LGL participated in the design of the study, participated in the data analysis and helped in drafting the manuscript. All authors read and approved the final manuscript.
